# Comparison of Non-Uniform Image Quality Caused by Anode Heel Effect between Two Digital Radiographic Systems Using a Circular Step-Wedge Phantom and Mutual Information

**DOI:** 10.3390/e24121781

**Published:** 2022-12-06

**Authors:** Ching-Ting Chang, Ming-Chung Chou

**Affiliations:** 1 Department of Medical Imaging, Kaohsiung Medical University Hospital, Kaohsiung 80708, Taiwan; 2 Department of Radiology, Kaohsiung Medical University Gangshan Hospital, Kaohsiung 82060, Taiwan; 3 Department of Medical Imaging and Radiological Sciences, Kaohsiung Medical University, Kaohsiung 80708, Taiwan; 4 Center for Big Data Research, Kaohsiung Medical University, Kaohsiung 80708, Taiwan; 5 Department of Medical Research, Kaohsiung Medical University Hospital, Kaohsiung 80708, Taiwan

**Keywords:** circular-step wedge, contrast-detail resolution, mutual information, visible ratio, anode heel effect

## Abstract

The purpose of this study was to compare non-uniform image quality caused by the anode heel effect between two radiographic systems using a circular step-wedge (CSW) phantom and the normalized mutual information (nMI) metric. Ten repeated radiographic images of the CSW and contrast-detail resolution (CDR) phantoms were acquired from two digital radiographic systems with 16- and 12-degree anode angles, respectively, using various kVp and mAs. To compare non-uniform image quality, the CDR phantom was physically rotated at different orientations, and the directional nMI metrics were calculated from the CSW images. The directional visible ratio (VR) metrics were calculated from the CDR images. Analysis of variance (ANOVA) was performed to understand whether the nMI metric significantly changed with kVp, mAs, and orientations with Bonferroni correction. Mann–Whitney’s U test was performed to compare the metrics between the two systems. Contrary to the VR metrics, the nMI metrics significantly changed with orientations in both radiographic systems. In addition, the system with the 12-degree anode angle exhibited less uniform image quality compared to the system with the 16-degree anode angle. A CSW phantom using the directional nMI metric can be significantly helpful to compare non-uniform image quality between two digital radiographic systems.

## 1. Introduction

Image quality is intimately related to the performance of disease diagnosis in medical images. In digital radiography, image quality can be quantitatively assessed by spatial resolution, contrast, and noise using the point/edge-spread function, modulation transfer function (MTF), and noise power spectrum (NPS), respectively [1,2,3]. However, none of these parameters can properly represent the overall image quality, and measurements of those metrics are relatively time-consuming and not suitable for routine practice in radiographic systems. The detective quantum efficiency (DQE) metric, which is a combined function of incident X-ray quanta per unit area, system gain, MTF, and NPS, is frequently used to measure the overall performance of radiographic systems [4,5,6]. However, DQE cannot reflect the entire imaging pipeline, such as image post-processing and corrections [7]. Although many studies have utilized contrast-detail resolution (CDR) phantoms to quantitatively evaluate the overall image quality of radiographic images [8,9,10,11,12], the asymmetric designs of these phantoms could not evaluate non-uniform image quality caused by the anode heel effect.

In radiographic systems, an X-ray tube design with a small anode angle has been clinically used to reduce the blurring effect and improve the sharpness of the object in radiographs, because the X-ray beam is produced with a small effective focal spot size on the anode. However, the small anode angle is known to induce the anode heel effect, where lower X-ray fluency and higher mean radiation energy are in the direction of the anode rather than the cathode [13]. The inhomogeneous distribution of X-ray photons is associated with non-uniformities in radiographic image quality; thus, previous studies have demonstrated that the signal-to-noise ratio (SNR) is lower in the direction of the anode of pelvic radiographs [14,15]. Because radiographic systems with different anode angles may exhibit different extents of the heel effect, and lower SNR in the anode direction may negatively influence clinical diagnosis for small and low-contrast lesions, it is important to understand how much percentage of image quality was decreased in the anode side and to compare the extent of non-uniform image quality between radiographic systems equipped with different anode angles.

To evaluate non-uniform image quality, a recent study proposed a circular step-wedge (CSW) phantom with normalized mutual information (nMI), and results its demonstrated that the directional nMI metric could successfully reflect the non-uniform image quality caused by the anode heel effect [16]. However, it remains unclear whether the CSW phantom was a suitable tool to compare the non-uniform image quality between radiographic systems equipped with different anode angles. Therefore, the purpose of this study was to quantitatively compare the non-uniform image quality between two radiographic systems with different anode angles using both CSW and CDR phantoms.

## 2. Materials and Methods

### 2.1. Image Acquisition

**Figure 1:**
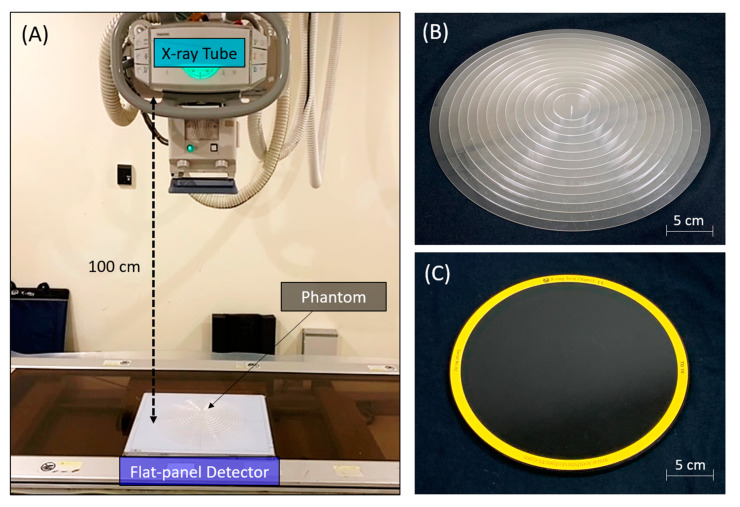
The experimental settings of the phantoms in a radiographic system. (**A**) The flat-panel detector was placed below the X-ray tube, and the source-to-detector distance was 100 cm. (**B**) The CSW phantom was designed with 14 step thicknesses and had a maximum diameter of 30 cm. (**C**) The CDR phantom was made with 144 circular details and had a diameter of 25 cm.

The experimental settings of the phantoms in a radiographic system are shown in Figure 1. The CSW phantom was made with acrylic materials with 14 step thicknesses increasing from 2 mm to 28 mm (incremental thickness = 2 mm), and with diameters decreasing from 30 cm to 4 cm (decremental diameter = 2 cm), as shown in Figure 1B [16]. The CDR phantom was made with 144 circular details, including 12 sizes × 12 contrasts (TO16, Leeds Test Objects LTD, North Yorkshire, UK; https://www.leedstestobjects.com accessed on 1 December 2022), as shown in Figure 1C [9]. Two digital radiographic systems (Toshiba DRX-1603B (DR-A) and Toshiba DRX-3724HD (DR-B), Tokyo, Japan) equipped with CsI thin-film-transistor flat panel detectors (CXDI-50C and CXDI-70C, Canon, Tokyo, Japan) and X-ray tubes with anode angles (16 and 12 degrees) were used. Both radiographic systems underwent regular calibration for X-ray output and flat-panel detection, such as detector gain, pixel defects, and inhomogeneity, by placing flat-panel detector 180 cm away from the X-ray tube. Ten repeated radiographic images of CSW and CDR phantoms were acquired with 40, 45, 50, 55, and 60 kV (at 5 mAs), and 0.5, 1, 2, 2.5, and 4 mAs (at 40 kVp), respectively. The matrix size and spatial resolution of the DR-A system were 2208 × 2688 and 0.16 × 0.16 mm^2^, respectively. The matrix size and spatial resolution of the DR-B system were 2800 × 3408 and 0.125 × 0.125 mm^2^, respectively. A dynamic range of 4096, source-to-detector distance of 100 cm, and field-of-view of 35 × 43 cm^2^ were kept identical for both systems. Furthermore, no heel effect correction was performed for any acquired images.

After image acquisition, all radiographic images were transferred to a standalone workstation. The CSW images were analyzed using a homemade script running on a MATLAB platform (Mathworks, Natick, MA, USA), while the CDR phantom images were analyzed using the commercial software AutoPIA (Leeds Test Objects LTD, North Yorkshire, UK). 

### 2.2. The Directional nMI Metrics from the CSW Phantom

**Figure 2:**
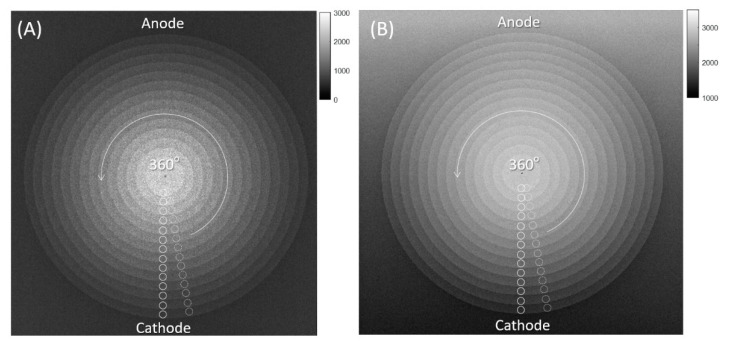
Directional nMI metrics from the CSW phantom. (**A**) A CSW image acquired on the DR-A (Toshiba DRX-1603B) system with an anode angle of 16 degrees. (**B**) A CSW image acquired on the DR-B (Toshiba DRX-3724HD) system with an anode angle of 12 degrees. Both images were acquired with 40 kVp and 0.5 mAs. The 14 circular ROIs were placed on the center of each step in one direction and were rotated 360 degrees about the center of CSW every 10 degrees.

For nMI metrics, the center of the CSW phantom was initially detected based on the center of gravity of the image. Second, 14 circular regions of interest (ROIs) were placed on the centers of the 14 steps in one direction. The nMI metric was then calculated based on the pixel values within the 14 ROIs, as described previously [16]. Specifically, each ROI contained N pixels, resulting in a total of 14*N pixel values that were used to calculate directional nMI metric. The information of 14 steps was defined as input values (*x* = 1, 2, 3, …, 14), whereas the 14*N pixel values were quantized into output values (*y* = 1, 2, 3, …, 4096). Subsequently, the MI was then calculated based on the definition of MI = H(*x*) + H(*y*) − H(*x*, *y*), where H(*x*) is the entropy of inputs, H(*y*) is the entropy of outputs, and H(*x*, *y*) is their joint entropy. As the maximum MI is dependent on the number of steps being used, the MI was divided by its maximum value to obtain nMI = MI/log_2_(14). In theory, the nMI is a quantitative measure that indicates how much information about input values can be conveyed by output values, so a higher nMI metric indicates higher similarity between the inputs and outputs and better image quality. Finally, the 14 ROIs were rotated counterclockwise every 10 degrees to obtain 36 directional nMI metrics from each CSW image, as shown in Figure 2.

### 2.3. The Directional VR Metrics from the CDR Phantom

**Figure 3:**
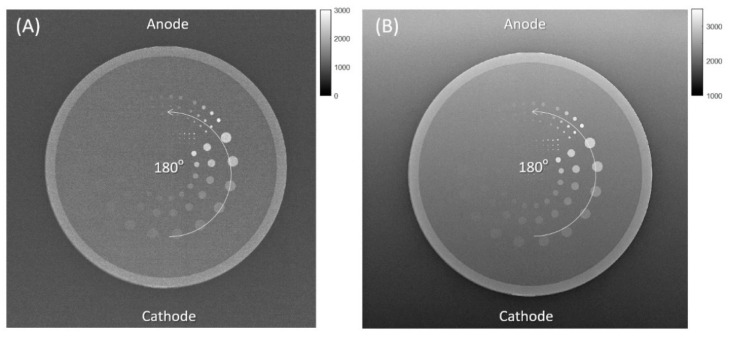
Directional VR metrics from the CDR phantom. (**A**) A CDR image acquired on the DR-A (Toshiba DRX-1603B) system with an anode angle of 16 degrees. (**B**) A CDR image acquired on the DR-B (Toshiba DRX-3724HD) system with an anode angle of 12 degrees. Both images were acquired with 40 kVp and 0.5 mAs. The phantom was physically rotated about the image center from 0 to 180 degrees every 30 degrees.

For VR metrics, we calculated the contrast-to-noise ratio (CNR) for each of the 144 details, defined as |detail signal–background signal|/(background noise), using the commercial software AutoPIA. The post-processing procedure is described in the software manual (https://www.cyberqual.it/ accessed on 1 December 2022). Second, the number of details whose CNR was higher than a default threshold was counted and was divided by the total number of details embedded in the phantom to generate a directional VR metric, defined as (number of detectable details)/(total number of details) [9]. Because the disc details are designed with different sizes and densities for detection, a higher VR metric reflects a higher detection rate and better image quality. Finally, the analysis was repeatedly performed for each CDR image acquired with orientations between 0 and 180 degrees, as shown in Figure 3.

### 2.4. Statistical Analysis

A one-way analysis of variance (ANOVA) was performed to understand how the nMI and VR metrics changed with kVp, mAs, and orientations in the two radiographic systems. A post hoc Wilcoxon signed-rank test was performed to compare the differences in nMI and VR metrics between two exposure parameters and between two orientations. In addition, a Mann–Whitney *U* test was performed to compare these metrics between two radiographic systems. The difference was considered statistically significant if *p* < 0.05 after Bonferroni correction to reduce type I error in multiple comparisons [17].

## 3. Results

**Figure 4:**
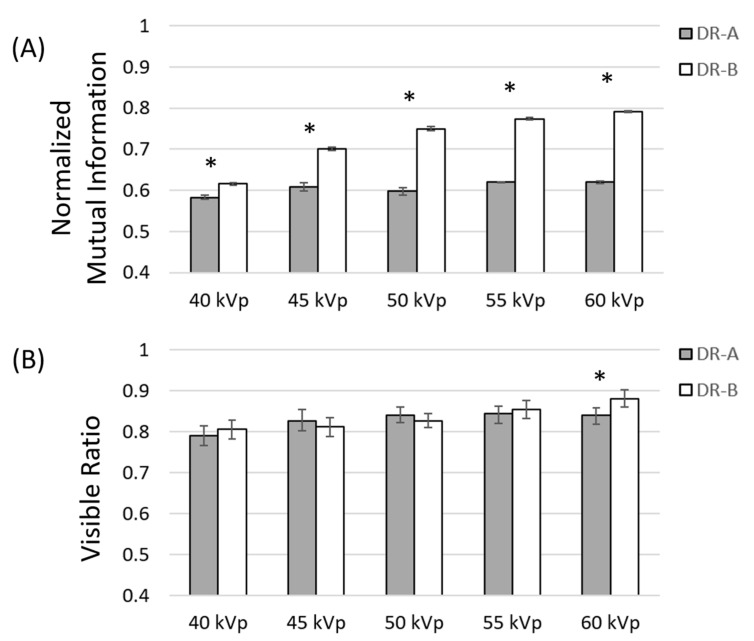
Comparison of normalized mutual information (**A**) and visible ratio (**B**) metrics at different kVp between two radiographic systems at 0.5 mAs. Asterisks indicate significant differences between the DR-A (Toshiba DRX-1603B) and DR-B (Toshiba DRX-3724HD) systems.

According to the mean values of directional nMI and VR metrics, our analysis showed that the overall image quality significantly changed between 40 and 60 kVp at a fixed 0.5 mAs. For the nMI metric, the post hoc comparison revealed significant differences between 40 and 60 kVp, as opposed to only 60 kVp in the VR metric, between the two systems, as shown in Figure 4.

**Figure 5:**
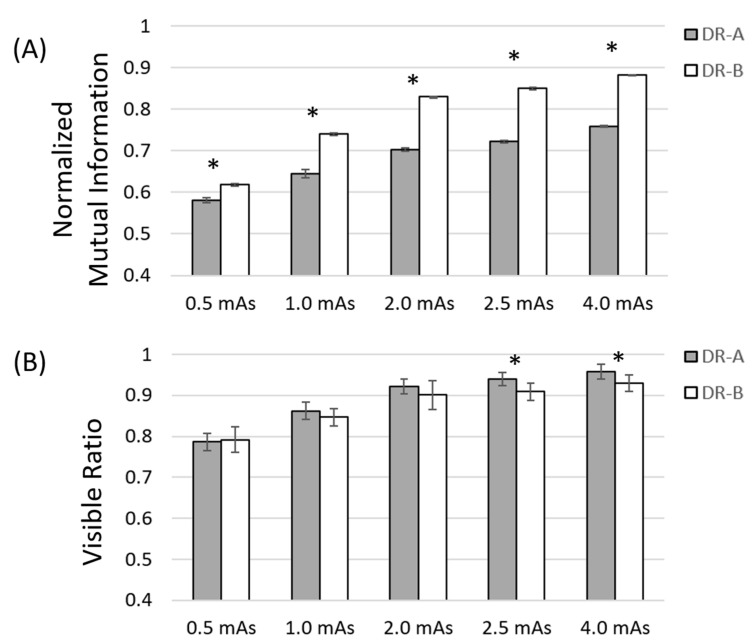
Comparison of normalized mutual information (**A**) and visible ratio (**B**) metrics at different mAs between two radiographic systems at 40 kVp. Asterisks indicate significant difference between the DR-A (Toshiba DRX-1603B) and the DR-B (Toshiba DRX-3724HD) systems.

Furthermore, our analysis demonstrated that the mean values of directional nMI and VR metrics significantly changed with mAs in the range between 0.5 and 4 mAs at fixed 40 kVp, and the post hoc comparison revealed a significant difference in nMI and VR metrics between any two mAs in the two radiographic systems. In addition, the nMI metrics were significantly different between the two radiographic systems at any mAs, whereas the VR metrics were significantly different between the two systems only at 2.5 and 4 mAs, as shown in Figure 5.

**Figure 6:**
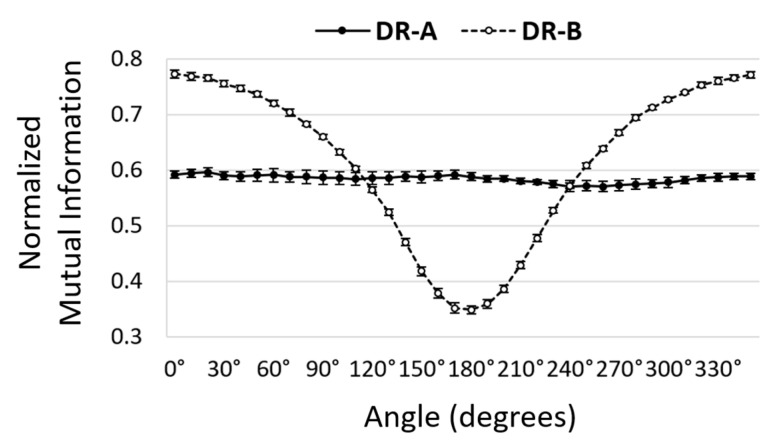
Comparison of directional normalized mutual information metrics at different angles between DR-A (Toshiba DRX-1603B) and DR-B (Toshiba DRX-3724HD) systems obtained using 40 kVp and 0.5 mAs. The nMI metrics were significantly different between the two systems in most orientations.

**Figure 7:**
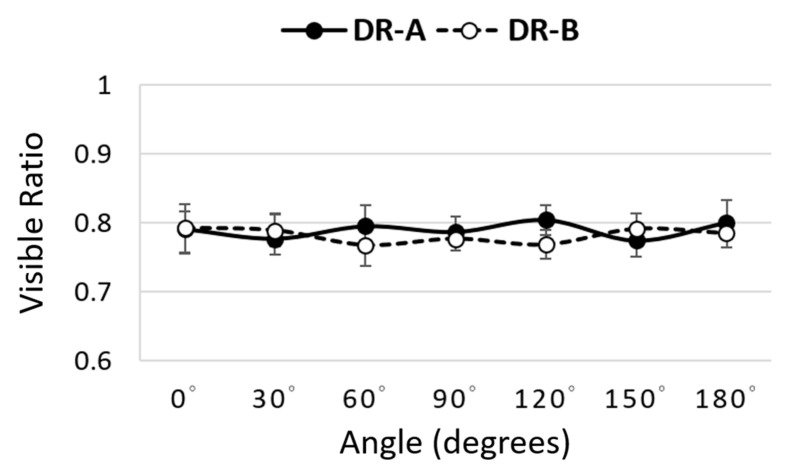
Comparison of directional visible ratio metrics at different angles between the DR-A (Toshiba DRX-1603B) and DR-B (Toshiba DRX-3724HD) systems obtained using 40 kVp and 0.5 mAs.

Our findings also revealed that the directional nMI metrics significantly changed according to the orientations 0°–180° in both radiographic systems. It was noted that the 16-degree anode angle in DR-A exhibited more uniform image quality than the 12-degree anode angle in DR-B, as shown in Figure 6. In DR-A, the directional nMI metrics were generally consistent across different angles, as opposed to DR-B, which were significantly lower at 180° (anode direction) compared to 0° (cathode direction) due to the heel effect. However, the directional VR metrics did not significantly change with orientations in either system, as shown in Figure 7.

## 4. Discussion

The small anode angle of the X-ray tube in radiographic systems has the advantage of creating a small effective focal spot that reduces blurring effects in radiographic images. However, the geometry of the anode heel with a smaller anode angle has a larger cross-sectional area to absorb the X-ray photons generated in the anode direction, causing inhomogeneous distribution of X-ray photons and non-uniform image quality. Previously, the MI metric was proposed to evaluate the image quality and was associated with imaging SNR, contrast, and resolution [18,19], and the directional nMI metric (CSW phantom) was capable of detecting the non-uniform image quality and outperformed the conventional VR metric (CDR phantom) in digital radiographs [16]. To the best of our knowledge, the nMI metric has not been utilized for quantitative comparisons of non-uniform image quality between two radiographic systems. The present study compared the directional nMI and VR metrics between two radiographic systems with different anode angles (16° and 12°) and showed that both metrics were significantly changed with kVp and mAs. Our results further revealed that contrary to the VR metrics, the directional nMI metrics were significantly changed with orientations. These findings support the previous study that a CSW phantom with a BMI metric was more suitable to evaluate non-uniform image quality compared to a CDR phantom with a VR metric [16].

The results of varying kVp and mAs demonstrated that both nMI and VR metrics increased significantly with kVp and mAs, suggesting that both metrics were able to reflect image quality in radiographic systems. The comparison of the two radiographic systems further showed that the DR-A system exhibited significantly lower nMI metrics than those of the DR-B system at different exposure parameters between 0.5–4 mAs and 40–60 kVp. However, the conventional VR metrics showed that the DR-A system exhibited significantly lower image quality than that of the DR-B system at 60 kVp (0.5 mAs) but exhibited significantly higher image quality than that of the DR-B system at 2.5 and 4 mAs (40 kVp). These findings suggested that the nMI was a more consistent metric to compare the image quality between different radiographic systems than the VR metric. Moreover, because the flat-panel detector (CXDI-70C) in the DR-B system incorporated a new Canon-developed glass substrate and had higher sensitivity than the old version of the flat-panel detector (CXDI-50C) in the DR-A system [20,21], the differences in image quality between the two radiographic systems might be attributable to different sensitivities between the two flat-panel detectors.

In addition, the results demonstrated that radiographic systems with a larger anode angle (16°) exhibited less variation in nMI metrics compared to smaller angles (12°), but no significant difference was noted in the VR metrics between two radiographic systems. The findings suggested that directional nMI metrics are more suitable to compare non-uniform image quality between two radiographic systems compared to the directional VR metrics. Although the system with the 16-degree anode angle exhibited a more uniform image quality, it had lower overall image quality compared to the system with the 12-degree anode angle. Consequently, it can be inferred that radiographic systems with larger anode angles exhibit more uniform image quality and are suitable for examination of large body parts, such as the chest and abdomen. By contrast, the radiographic systems with smaller anode angles can produce a small focal spot and reduce blurring and are suitable for the examination of small body parts, such as wrist and finger. Furthermore, the higher image quality on the cathode side can be further utilized to detect small and low-contrast lesions. Therefore, directional nMI metrics may help radiographers optimize image quality by positioning the body part of interest within the FOV in different radiographic systems.

In the CDR phantom, the centralized design of the 144 embedded disk details rendered the VR metric insensitive to non-uniformities across the entire FOV. However, the disk details are not symmetrically arranged, and it was necessary to rotate the phantom to evaluate the directional image quality in multiple acquisitions. In contrast, the symmetrically circular fashion of the CSW phantom allowed us to evaluate directional image quality in one acquisition, and thus it can be used to efficiently compare non-uniform image quality between many radiographic systems. Moreover, the acrylic material used in the CSW phantom could more sensitively detect changes in image quality than the CDR phantom [12].

This study had some limitations. First, the exposure parameters used in this study ranged between 0.5–4 mAs and 40–60 kVp, due to the thin thickness of phantoms. Second, our hospital only has radiographic systems with two kinds of anode angles (12 and 16 degrees), so the present study chose two representative radiographic systems for comparisons. However, further investigation will be needed to compare other radiographic systems with distinct anode angles. Third, the nMI metric is less sensitive to variations in spatial resolution [18]. Both radiographic systems had similar spatial resolution (0.16 vs. 0.125 mm), and thus our results may not reflect differences in spatial resolution. Finally, the CDR phantom was only placed at the central FOV with different orientations. However, the peripheral FOV may have more variations in X-ray fluency than the central FOV, suggesting that the VR metric may change with distance from the central FOV.

## 5. Conclusions

In conclusion, the present study compared the non-uniform image quality caused by the anode heel effect between two radiographic systems with different anode angles using both nMI and VR metrics. The results showed that the nMI metric was a more stable metric to compare the image quality between two radiographic systems than the conventional VR metric at different exposure parameters. Further, the comparison demonstrated that the nMI metric was more sensitive in detecting changes in image quality caused by the heel effect than the VR metric and that a radiographic system equipped with a larger anode angle exhibited more uniform image quality than that with a smaller angle. Therefore, we concluded that the directional nMI metric with a CSW phantom is a suitable and convenient tool to compare non-uniform image quality caused by heel effect between two radiographic systems with different anode angles.

## Data Availability

Not applicable.
